# Reduced translation efficiency due to novel splicing variants in 5′ untranslated region and identification of novel cis-regulatory elements in canine and human *BRCA2*

**DOI:** 10.1186/s12860-020-00336-4

**Published:** 2021-01-06

**Authors:** Yasunaga Yoshikawa, Hajime Kozuma, Masami Morimatsu, Kaori Sugawara, Koichi Orino

**Affiliations:** 1 Laboratory of Veterinary Biochemistry, School of Veterinary Medicine, Kitasato University, Aomori, 034-8628 Japan; 2 Laboratory of Laboratory Animal Science and Medicine, Department of Disease Control, Graduate School of Veterinary Medicine, Hokkaido University, Sapporo, 060-0818 Japan

**Keywords:** BRCA2, Expression level, Splicing variant, Translation efficiency, 5′ untranslated region

## Abstract

**Background:**

Breast cancer 2, early onset (*BRCA2*) is a tumor suppressor gene. The protein encoded by this gene plays an important role in homologous recombination (HR)-mediated DNA repair. Deleterious mutations in *BRCA2* and downregulation of its expression have been associated with tumorigenesis in dogs and humans. Thus, regulation of BRCA2 expression level is important for maintaining homeostasis in homologous recombination.

**Results:**

In this study, the mechanisms that regulate the expression of *BRCA2* were proposed. Novel splicing variants were identified in the 5′ untranslated region (UTR) of canine and human *BRCA2* in canine testis, canine ovary, and canine and human cultured cell lines. In cultured cells, the ratio of *BRCA2* splicing variants at the 5′ UTR was altered by serum starvation. These novel splicing variants, excluding one of the canine splicing variants, were found to reduce the translational efficiency. Additionally, the DNA sequence in human *BRCA2* intron 1 harbored novel cis-regulatory elements. Three silencer and two enhancer cis-regulatory elements were identified in human *BRCA2* intron 1.

**Conclusions:**

This study demonstrates that BRCA2 expression level is regulated via 5′ UTR splicing variants and that the *BRCA2* intron 1 region harbors cis-regulatory elements.

## Background

Breast cancer 2 early onset (*BRCA2*) is a tumor suppressor gene encoding a protein that contributes to homologous recombination (HR)-mediated DNA repair [1]. As BRCA2 plays a crucial role in DNA repair, deleterious mutations in this gene have been reported to be associated with tumorigenesis in dogs and humans [2, 3]. Our previous studies demonstrated that mutation and downregulation of *BRCA2* were both associated with canine mammary tumors [4, 5]. We identified loss of heterozygosity as an underlying mechanism by which BRCA2 expression levels are reduced in canine mammary tumors [6]. A recent study has revealed how haploinsufficiency is induced by *BRCA2* mutation [7]. Thus, downregulation of *BRCA2* is one of the causes of tumorigenesis. In cells, reduced or excess *BRCA2* expression creates an imbalance between *BRCA2* and *RAD51.* RAD51 is a DNA recombinase that interacts with BRCA2 and is recruited to DNA damage sites to carry out strand invasion, which is a vital step of homologous recombination. The imbalance between these gene products affects HR activity [8, 9]. These studies suggest that regulation of BRCA2 expression level is important for maintaining the homeostasis of HR.

BRCA2 also contributes to HR in meiosis, and its expression level is higher in the gonads than in other tissues. In germ cells, BRCA2 interacts with RAD51 and DMC1, which are meiosis-specific DNA recombinases, and recruits them to the recombination site to initiate strand exchange [10]. In mice, insufficiency of BRCA2 leads to infertility, mostly because of failures in recombination in germ cells [11]. Thus, the expression level and function of BRCA2 is vital in germ cells, and regulating the BRCA2 expression level is important for meiosis and maintaining DNA integrity. However, in humans, relationships between *BRCA2* mutations and fertility have been controversial [12–14].

Several mechanisms have been reported to regulate BRCA2 expression levels. For example, the transcription factor nuclear factor-κB transactivates BRCA2 and p53. ZAR1L and PARP1 were reported as repressors of BRCA2 [15–18]. Several microRNAs have been reported to downregulate BRCA2 expression. The overexpression of these microRNAs have been identified to be associated with tumorigenesis [19, 20]. At the protein level, BRCA2 is ubiquitinated by USP11 to undergo subsequent degradation [21]. Although several factors are involved in regulating BRCA2 expression, the underlying mechanisms still remain unclear. Another mechanism that could be regulating the expression is the production of splicing variants at the 5′ untranslated region (UTR). Alternative splicing has been previously reported to play an important role in the regulation of gene expression [22]. It is thought that the alternative start codons produce unexpected translation products that reduce the levels of primary transcripts. Splicing variants at the 5′ UTR also reduce translational levels by forming a complex and bulky secondary structure that inhibits ribosome sliding.

In this study, we identified novel splicing variants at the 5′ UTR of canine *BRCA2* in ovary and testis samples. These splicing variants were also expressed in cultured cells from humans and dogs. The expression levels varied according to the condition of the cells. We also demonstrated that these 5′ UTR splice variants affected the translation efficiency. Surprisingly, the 5′ UTR sequences of canine and human *BRCA2* were also shown to play a role in the regulation of transcriptional activity. This is the first study to demonstrate that BRCA2 expression level is regulated by 5′ UTR splicing variants. We also show that the intron 1 region harbored cis-regulatory elements.

## Results

### A novel exon was identified between canine *BRCA2* exons 1 and 2

**Fig. 1:**
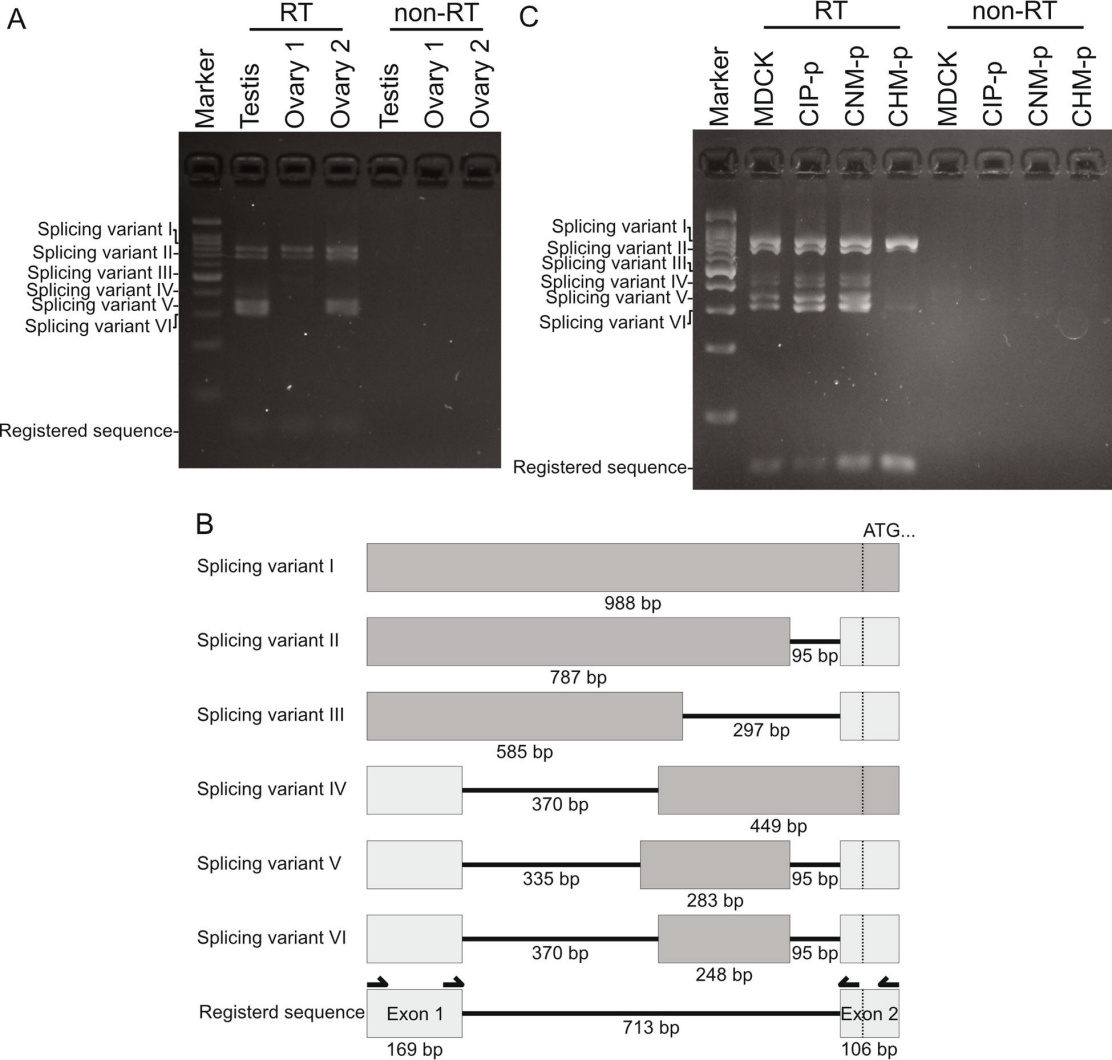
Identification of novel splicing variants in canine *BRCA2* in ovary and testis. **a** RT-PCR products around the canine *BRCA2* intron 1 in two ovary and one testis samples were electrophoresed. Splicing variants, referred to as splicing variant I-VI and registered sequence are indicated. **b** Diagram of novel splicing variants in canine *BRCA2* intron 1. Arrows indicate primers using RT-PCR and nested-PCR. **c** RT-PCR products from canine cell lines were electrophoresed and splicing variants excluding splicing variant III were detected. RT indicates RT-PCR products and non-RT indicates PCR products without reverse transcription. Arrows indicate primers used in RT-PCR and nested-PCR

We identified a novel exon between canine *BRCA2* exons 1 and 2 via RT-PCR using samples from two ovaries and one testis (Fig. 1 a). These PCR products were sequenced to determine their splicing patterns (GenBank accession no. LC547963, LC547964, LC547965, LC547966, LC547967 and LC547968). We identified six splicing variants in addition to the annotated transcript (Fig. 1 b). These variants were identified between originally reported exon 1 and exon 2 in 5′ UTR of canine *BRCA2* gene and complied with the GU-AG rule (Supplemental Fig. 1). These splice variants resulted in mRNA transcripts that varied in size from 248 bp to 988 bp. We referred to the splicing variants as I-VI, I being the variant that encodes the longest transcript and VI being the shortest. Novel splicing variant I contained 8 possible start codons, which potentially translate short and truncated proteins, and splicing variant VI, the shortest variant, contained 5 possible start codons, whereas the registered sequence does not have a start codon in the 5′ UTR (Supplemental Fig. 1). It is not easy to determine the precise expression level by RT-PCR, but splicing variants I, II, V, VI and registered sequence were the main transcripts (Fig. 1 a). We also tested the expression of these splicing variants in cultured cells derived from dogs. All four cell lines showed the presence of these spliced variants, similar to testis and ovary tissues (Fig. 1 c) and the splicing variants I, II, V, VI and the registered variant showed higher mRNA levels.

### Expression pattern and levels of splicing variants in cultured cells were affected by serum starvation

**Fig. 2:**
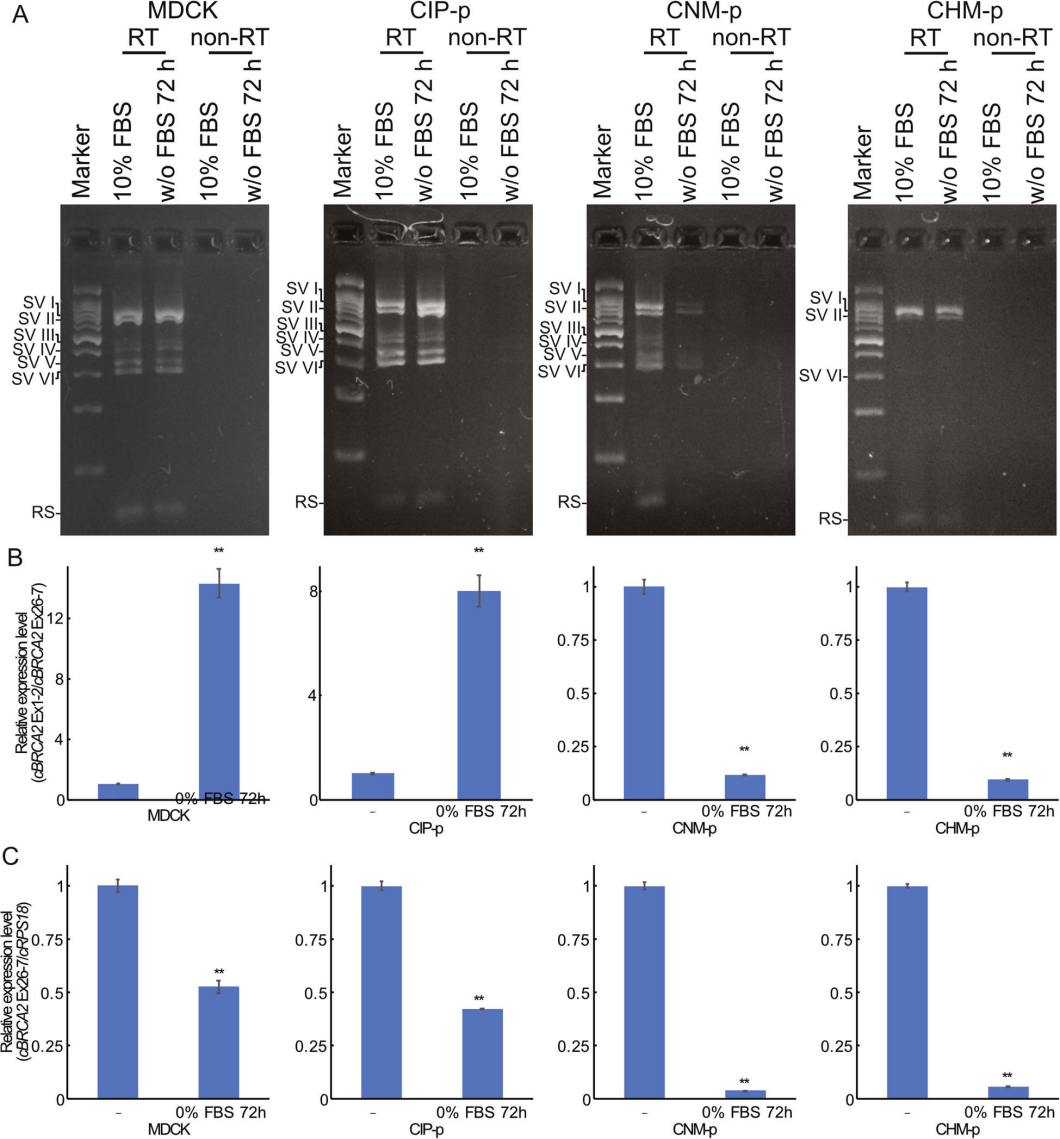
Serum starvation effects on expression pattern and ratio of *BRCA2* 5′ UTR splicing variants. **a** RT-PCR products between canine *BRCA2* exon 1 and exon 2 in four canine cell lines with or without 72-h serum starvation were electrophoresed. RT indicates RT-PCR products and non-RT indicates PCR products of the templates without reverse transcription. SV and RS indicate splicing variant and registered sequence, respectively. **b** Relative expression ratio of registered sequence (ordinary exon 1–2) versus total canine *BRCA2* expression (exon 26–27) in four canine cell lines was measured by quantitative PCR. To quantify registered sequence, the exon 1 and 2 spanning primer was used. **c** Relative expression level of canine *BRCA2* normalized by canine *RPS18* in four canine cell lines. “**” indicates *p* < 0.01

The expression level of BRCA2 is regulated by the cell cycle and the condition of the cells. Thus, we tested the effect of serum starvation, which introduced G1 phase arrest, on cultured cell lines. The expression pattern of splicing variants in CHM-p cells changed due to serum starvation. However, MDCK and CIP-p cells maintained the same expression pattern even after serum starvation (Fig. 2 a). In CNM-p cells, expression levels of splicing variants III and IV and the registered sequence were reduced after serum starvation. In CHM-p cells, levels of splicing variants VI and the registered sequence were reduced after serum starvation (Figs. 1 and 2 a). CNM-p and CHM-p cells also showed relatively increased expression levels of splicing variant II compared to splicing variant I upon serum starvation (Fig. 2 a). In all the four cell lines mentioned above, the expression level of BRCA2 was reduced by serum starvation (Fig. 2 b). The relative ratio of expression levels of the BRCA2 variants with the shortest registered sequence to total BRCA2 was also changed (Fig. 2 c). MDCK and CIP-p cells showed an increased relative expression ratio of registered sequence, but it was decreased in the cases of CNM-p and CHM-p (Fig. 2 c).

### Human cultured cell lines also expressed similar splicing variants

**Fig. 3:**
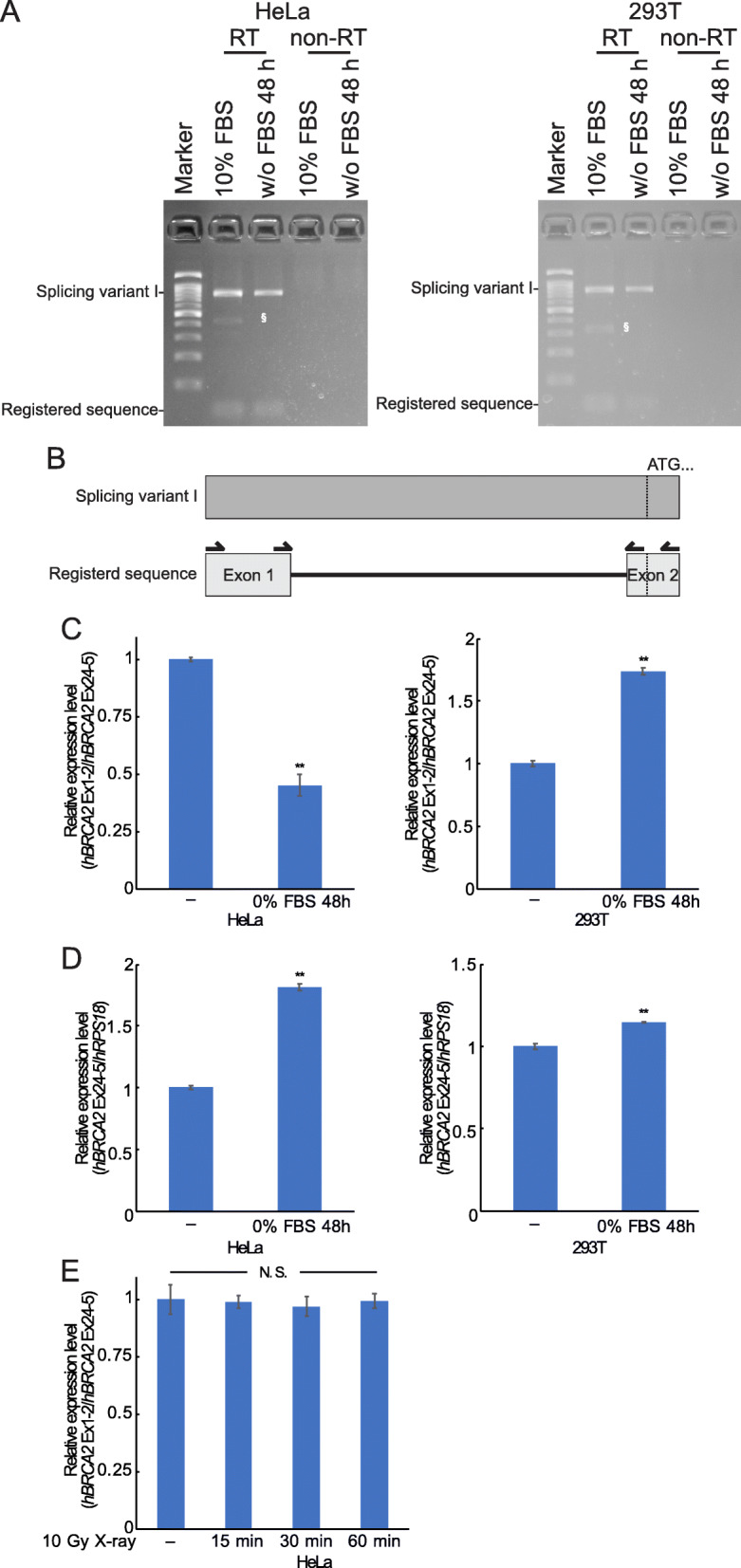
Human cell lines, HeLa, and 293 T cells also expressed splicing variants. **a** RT-PCR products between human BRCA2 exon 1 and exon 2 in HeLa and 293 T cell lines with or without 48-h serum starvation were electrophoresed. RT indicates RT-PCR products and non-RT indicates PCR products using the templates without reverse transcription. Arrows indicates primers using RT-PCR and nested-PCR. “§” indicates PCR products with first PCR primers. **b** Diagram of novel splicing variants in human *BRCA2* intron 1. Arrows indicate primers used in RT-PCR and nested-PCR. **c** Relative expression ratio of registered sequence (ordinary exon 1–2) versus total human *BRCA2* expression (exon 25–26) in HeLa and 293 T cells was measured via quantitative PCR. To quantify the level of the registered sequence, the exon 1 and 2 spanning primer was used. **d** Relative expression level of human *BRCA2* normalized using human *RPS18* in HeLa and 293 T cells. **e** HeLa cells treated with 10 Gy X-rays were harvested at the indicated time points after irradiation. The relative expression ratio of registered sequence (ordinary exon 1–2) versus total human *BRCA2* expression (exon 25–26) was measured as in Fig. 3 (**c**). “**” indicates *p* < 0.01. “N. S.” indicates not significant

Canine tissues and cultured cells expressed splicing variants in canine *BRCA2* 5′ UTR, and the expression ratio of these variants was regulated by the condition of the cells. We hypothesized that regulation of *BRCA2* gene expression via splicing variants could also be present in humans. We tested this using two human derived cell lines, HeLa and 293 T. Splicing variants including the entire intron 1 sequence were expressed in these cell lines (Fig. 3 a and b, GenBank accession no. LC547969). The novel splicing variant contained 9 possible start codons, which potentially translate short and truncated proteins, whereas the registered sequence did not have a start codon in the 5′ UTR (Supplemental Fig. 1). Although the expression pattern of the splicing variants was not changed by serum starvation, the relative ratio of registered sequence and shorter splicing variant was reduced or increased with a slight increase in BRCA2 expression level in HeLa or 293 T cells (Fig. 3 c). We also tested the effect of X-ray irradiation, as some genes have been reported to change the ratio of splicing variants to regulate expression level in response to DNA damage [23, 24]. However, the relative ratio of the registered sequence did not show any drastic change until 60 min after 10 Gy X-ray treatment (Fig. 3 e).

### Novel splicing variants of canine and human BRCA2 suppressed translational efficiency

**Fig. 4:**
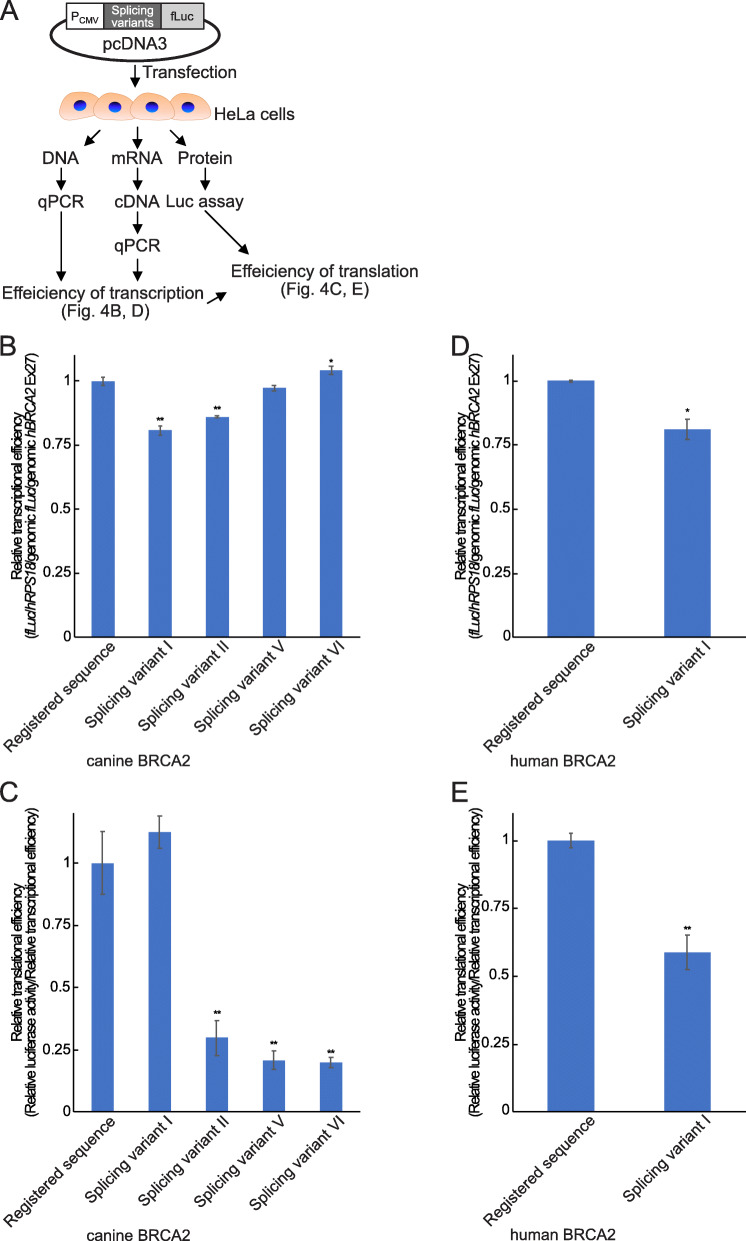
Effect of novel splicing variants on transcription/translation activity in cells. **a** Schematic diagram depicting how the transcriptional and translational activity were measured. Firefly luciferase expression vectors with each splicing variant and *Renilla* luciferase expression vector were transfected to HeLa cells followed by DNA, mRNA, and protein extraction after 48 h. The relative expression level of firefly luciferase mRNA was measured through quantitative PCR normalized with the house-keeping gene human *RPS18* and the transfection efficiency by genomic luciferase and human *BRCA2* exon 27 region from DNA samples. To determine the efficiency of translation activity, firefly and *Renilla* luciferase activity were detected using dual-luciferase reporter assay normalized by transcriptional activity. **b** Relative transcriptional efficiency of canine *BRCA2* splicing variants containing luciferase vector. **c** Relative translational efficiency of canine *BRCA2* splicing variants containing luciferase vector. **d** Relative transcriptional efficiency of human *BRCA2* splicing variants containing luciferase vector. **c** Relative translational efficiency of human *BRCA2* splicing variants containing luciferase vector. “**” indicates *p* < 0.01

The novel splicing variants of BRCA2 were expressed in canine tissues and both canine and human derived cell lines and these ratios were changed by the conditions in which the cells were maintained. We speculated that these splicing variants regulate the translational efficiency of BRCA2. To evaluate the translational efficiency of BRCA2, luciferase assay with quantitative PCR were employed (Fig. 4 a). To evaluate the translational efficiency, we designed plasmids containing CMV promoter followed by each 5′ UTR variant sequence and luciferase. These constructs might also contribute to the suppression of the transcriptional efficiency. Thus, to avoid this effect, we quantified the expression level of firefly luciferase and the transfection efficiency of the plasmid DNA construct containing firefly luciferase gene and genomic DNA of human BRCA2 exon 27 was determined via quantitative PCR. In canine *BRCA2*, the splicing variants, I, II, V, VI, and the registered sequence were the major variants in tissues and cell lines. Thus, we evaluated these five variants. Figure 4 b and d shows the relative expression level of firefly luciferase. Unexpectedly, splicing variants I and II of canine *BRCA2* and splicing variant I of human *BRCA2* suppressed the CMV promoter activity (Fig. 4 b and d). In parallel, luciferase assay was performed to evaluate the translational activity. The values of the transcriptional activity were used to normalize the relative translational activity. The splicing variants of canine *BRCA2* excluding splicing variant I and splicing variant I of human *BRCA2* were found to be associated with decreased translational activity (Fig. 4 c and e).

### Intron 1 of canine and human *BRCA2* also suppressed *BRCA2* promoter activity

**Fig. 5:**
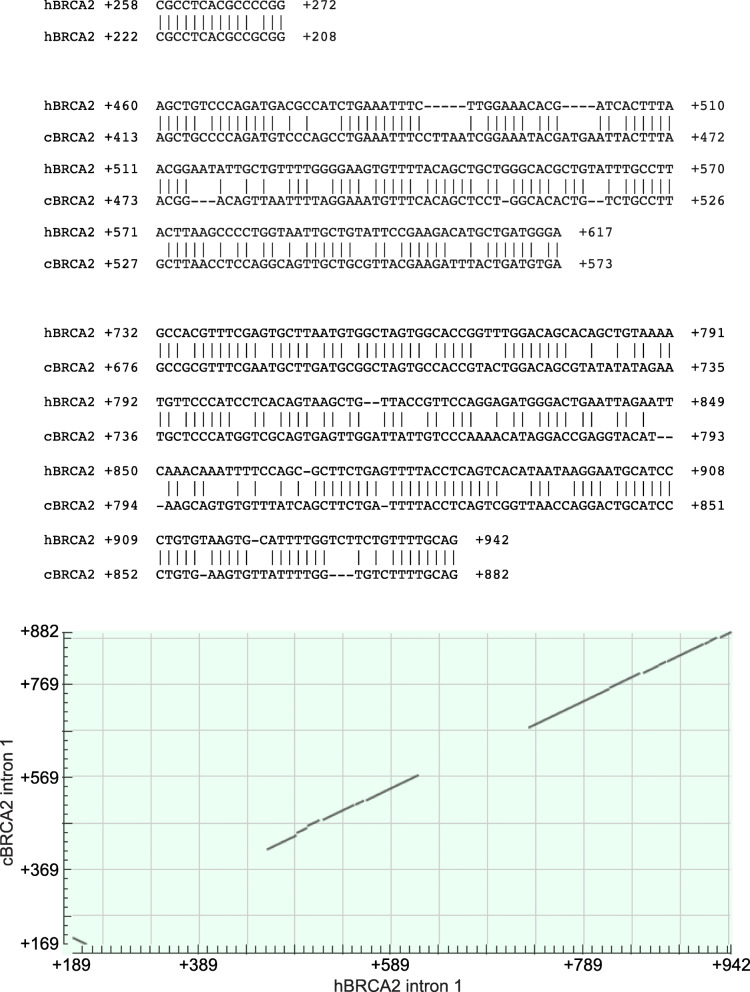
Alignment of canine and human *BRCA2* intron 1. Canine and human *BRCA2* intron 1 were aligned by BLAST (https://blast.ncbi.nlm.nih.gov/Blast.cgi). Three regions are conserved between canine and human *BRCA2* intron 1. The lower panel shows a dot plot view of the alignment

**Fig. 6:**
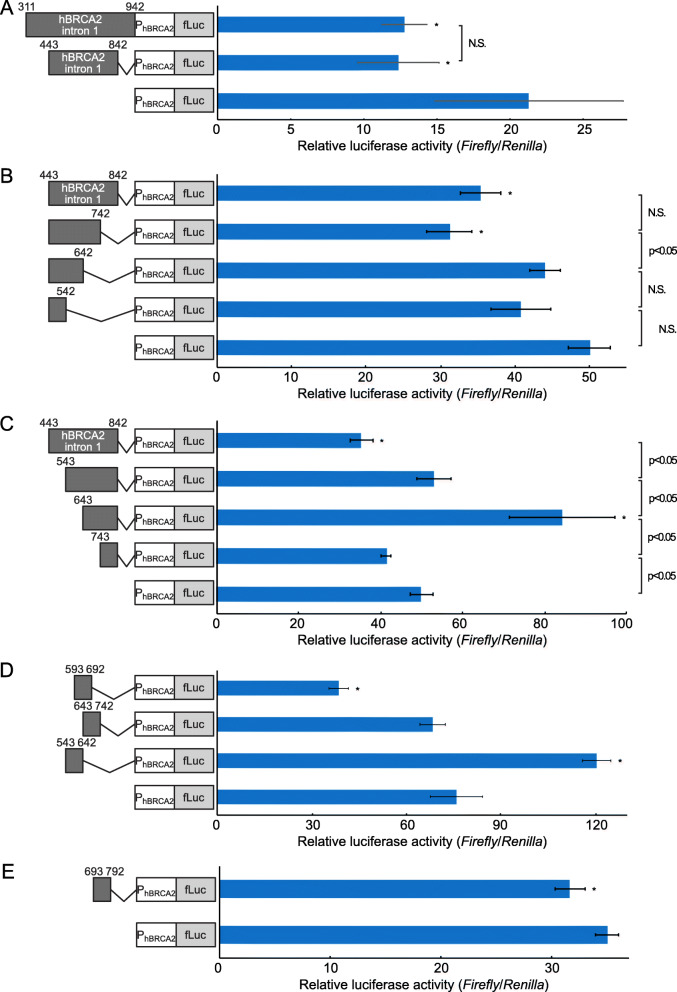
Effect of human *BRCA2* intron 1 region on transcription activity. Human *BRCA2* intron 1 region was inserted upstream of human *BRCA2* promoter region (− 187 − + 310 bp) to measure the transcriptional activity using dual-luciferase reporter assay. **a** The promoter activities of the entire intron 1 region without promoter (+ 311 − + 942 bp) and conserved central 500 bp (+ 443 − + 842 bp) were measured. The promoter activities of sequential 100 bp deletion from 3′ end (**b**) and 5′ end (**c**) were compared. **d** and **e** To analyze in more detail the cis-regulatory elements in human *BRCA2* intron 1, the indicated 100 bp of human *BRCA2* intron 1 was inserted upstream of human *BRCA2* promoter and compared. “*” indicates *p* < 0.05 compared with the construct that only had the *BRCA2* promoter. “N. S.” indicates not significant.

Novel splicing variants of canine and human *BRCA2* suppressed CMV promoter activity in addition to the translational suppression. Thus, we next tested the effect of the DNA sequence of various splicing variants on *BRCA2* promoter activity. Prior to this, canine and human *BRCA2* intron 1 regions were compared to find conserved DNA sequences, because the novel exon region identified in intron 1 was different from the registered sequence and conserved sequence had the greatest potential to be a silencer element (Fig. 5). Three conserved regions were identified in canine and human *BRCA2*. A conserved 15 bp 5′-terminal region was located in the *BRCA2* promoter. The other two regions, human *BRCA2* + 460 − + 617 bp and + 732 − + 942 bp, were also found to be conserved within canine *BRCA2* intron 1. Thus, we expected these two conserved DNA sequences to have a silencer element. Because canine *BRCA2* splicing variant II, which did not have 3′-terminal 95 bp of intron 1, still suppressed the effect of CMV promoter activity, thus the sequence around + 460 − + 841 bp in human *BRCA2* (corresponding + 413 − + 787 bp in canine *BRCA2*) should also be associated with silencer elements. To avoid translational effects, the human *BRCA2* intron 1 region without promoter sequence (− 187 − + 310 bp) was inserted upstream of human *BRCA2* promoter (Fig. 6 a). As expected, + 443–843 bp region in human *BRCA2* decreased the promoter activity similar to human *BRCA2* intron 1 region (+ 311–942 bp). Next, we attempted to identify where the silencer element was located within the 500 bp sequence. Sequential deletion mutants in 5′ and 3′ ends were compared with the promoter activity (Fig. 6 b and c). The + 743 − + 842 bp region contained a cis-regulatory silencer element (Fig. 6 c). The region of + 443 − + 542 bp also contained a silencer, as the presence of this region showed significantly reduced promoter activity than that of the + 543 − + 842 bp region (Fig. 6 c) and the + 443 − + 542 bp region tended to reduce the promoter activity but it was not significant (Fig. 6 b; *p* = 0.0295, by Holm’s method; *p* value < 0.0125 was considered significant.). Unexpectedly, the region around + 643 − + 742 bp harbored an enhancer element, and the + 543 − + 642 bp region contained a silencer element (compare Fig. 6 c to Fig. 6 b). Thus, we speculated that cis-regulatory elements could be separated in some constructs. To study this region in detail, three distinct constructs were tested (Fig. 6 d). As we expected, the + 593 − + 692 bp region contained a silencer element. However, surprisingly, the + 543 − + 642 bp region acted as an enhancer element. To identify the enhancer elements around + 742 bp in intron 1, we also analyzed the + 693 − + 792 bp region (Fig. 6 e). This region showed slightly decreased *BRCA2* promoter activity. Thus, we concluded that the silencer and enhancer elements were too close to be separated, and the effect of the silencer element in this region was stronger than the enhancer element.

## Discussion

BRCA2 expression levels must be tightly regulated, in particular to maintain a balance between the expression level of BRCA2 and RAD51, which is required for effective HR [9, 25]. Here, we identified a new translation regulation mechanism in both canine and human BRCA2, which is mediated by splicing variants at the 5′ UTR that have been reported to usually regulate gene expression [22]. All canine *BRCA2* splicing variants excluding splicing variant I and human *BRCA2* splicing variant I decreased translational efficiency.

The expression ratios of these splicing variants were altered by the condition of the cells. In CNM-p and CHM-p canine cultured cells, the ratio of transcripts without the intron 1 region compared to total BRCA2 was decreased after serum starvation, which induced G1 phase arrest. Especially in CNH-p and CHM-p cells, the splicing variant pattern was also shifted to favor the long form after serum starvation. Splicing variants containing the intron 1 region, excluding canine splicing variant I, showed decreased translational activity. Thus, in these cells, BRCA2 protein levels were decreased via suppression of translation. MDCK and CIP-p canine cells demonstrated an increased ratio of splicing variants without the intron 1 region after serum starvation. In addition to this, the decreased level of *BRCA2* mRNA in these cells was relatively not as significant as in case of CNM-p and CHM-p cells. Thus, these cells seemed to maintain the BRCA2 protein level after serum starvation. Like canine cell lines, there were two types of responses in human cells. In HeLa cells, the ratio of transcripts without the intron 1 region versus total BRCA2 was decreased, whereas it increased in 293 T cells. Splicing variant I of human BRCA2 showed decreased translational activity. Thus, in HeLa cells, the BRCA2 protein level was decreased via suppression of translation, but not increased in 293 T cells. However, why there is a difference in response is not clear. We speculated that the sensitivity to the serum starvation was associated with *BRCA2* expression level and splicing variant ratio, because CNM-p and CHM-p cells were more sensitive to serum starvation than MDCK cells (data not shown). We also speculated that these splicing variants contributed to the protein expression after X-ray irradiation, as greater amounts of transcripts of splicing variants were induced in response to DNA damage [23, 24]. However, the ratio of splicing variants was not altered by irradiation, thus the novel splicing variant of BRCA2 at 5′ UTR was not affected by DNA damage.

The mechanisms underlying the suppression of expression by these novel splicing variants are unknown, but there are two possibilities based on the reports on the regulation of other genes [22]. First, regulation may occur through the upstream start codon in the 5′ UTR. The upstream start codons translate short or truncated proteins and prevent the production of the main open reading frame products [22]. Each novel splicing variant of canine *BRCA2* possesses 5–8 start codons which potentially translate short peptides or truncated proteins. As the reference sequence does not contain a start codon in the 5′ UTR, these upstream start codons in the 5′ UTR potentially prevent translation efficiency of the primary open reading frame. The second possibility involves the RNA secondary structure. Both novel splicing variants showed complex and bulky structures according to a secondary RNA structure prediction program. This type of structure obstructs ribosome sliding and scanning. Some genes are regulated by the RNA secondary structure of splicing variants at the 5′ UTR [22]. Although splicing variant I of canine *BRCA2* still possessed similar translational activity compared to the registered sequence, canine and human *BRCA2* are potentially regulated by these mechanisms.

The sequence of the splicing variants affected transcription efficiency as well as translation. Thus, canine and human intron 1 regions in the genomic DNA act as cis-regulatory elements. A few studies have described *BRCA2* promoter silencer elements, but this is the first study to show that this region, which is a part of the *BRCA2* intron 1, functions as cis-regulatory elements [15–17, 26]. First, we expected that there had been only a silencer element in intron 1, but our thorough analysis indicated that this region consisted with at least three silencer elements and two enhancer elements. Supporting this notion, in human *BRCA2* intron 1, accumulation of histone H3K27Ac, which indicates the presence of active promoter and enhancer regions, were reported by the UCSC genome browser (https://genome.ucsc.edu). We did not investigate which trans-regulatory elements interact with this novel cis-regulatory element. This point will be the next important research issue to understand how *BRCA2* expression is regulated.

Although *BRCA2* was identified more than a twenty-five year ago and many studies of this gene have been reported in humans, canine and mice, the regulatory system identified in this study has not been previously reported. Both canine and human *BRCA2* had similar splicing variants, thus it seemed that mammalian BRCA2 employs splicing variants at the 5′ UTR as an expression regulation system. Parts of the intron 1 region were conserved between human and canine, thus transcriptional regulation by the novel cis-regulatory element also seemed to be conserved in mammals. These points are interesting when considering the evolution of BRCA2.

## Conclusions

In this study, we identified novel splicing variants at the 5′ UTR of canine and human *BRCA2* in canine ovary and testis and canine and human cell lines. These splicing variants reduced the translation efficiency of the gene. The expression ratio of splicing variants was altered depending on the conditions in which the cells were maintained. Unexpectedly, the intron 1 sequence in *BRCA2* also suppressed transcription efficiency. This led to the identification of silencer and enhancer cis-regulatory elements in *BRCA2* intron 1 region. Although further studies are needed to determine how the splicing variants regulate translation and how novel cis-regulatory elements affect transcription, our data suggests that the BRCA2 expression level was regulated by the novel splicing variants at the 5′ UTR and cis-regulatory elements in intron 1 in canine and human.

## Methods

### Cell culture

All cultured cells were grown in 100 mm plastic dishes containing 10 mL of Dulbecco’s modified Eagle’s medium supplemented with 10% fetal bovine serum at 37 °C under humidified 5% CO_2_–95% air.

### RNA extraction for reverse-transcription PCR

Two ovary and one testis samples from three 1-year-old mixed breed dogs were obtained from the Honda Animal Hospital. This sampling was done as part of contraception or castration under general anesthesia by veterinarians. These tissue samples in RNAlater solution (Life Technologies, Carlsbad, CA, USA) and all cultured cell pellets were stored at − 80 °C until they were processed for RNA extraction using TRIzol RNA Isolation Reagents (Life Technologies, Carlsbad, CA, USA) as previously described [27]. All experimental procedures were approved by and conducted in accordance with the Guidelines for Institutional Laboratory Animal Care and Use of the School of Veterinary Medicine at Kitasato University, Japan (Approval Number: 11–065).

### Reverse-transcription-PCR and nested PCR

Reverse-transcription of RNA were performed using SuperScript™ IV VILO™ Master Mix (Life Technologies, Carlsbad, CA, USA) according to the manufacturer’s instructions. To amplify canine and human *BRCA2*, each reaction mixture contained 100 ng of genomic DNA as a template, and 300 nM forward and reverse primers (Table 1), and KOD One PCR master mix (Toyobo, Osaka, Japan) was used with a total volume of 100 μL. PCR conditions included one cycle of initial denaturation for 10 s at 98 °C, followed by 25 cycles with 10 s of denaturation at 98 °C, 25 s of annealing and elongation at 68 °C, and a final extension step of 1 min at 68 °C. The PCR products were diluted 100–2000 times and PCR was performed with same conditions using nested-PCR primer sets. The PCR products were sequenced to identify the splicing variants.

**Table 1:** Base sequences of primers used in this study

	Forward primer	Reverse primer
For amplification of 5′ UTR		
Canine BRCA2 first PCR	5′-gggggatccAAAGAAGGTCGGCGGAGGCG-3′	5′-gggctcgagCTGCTTGATTGCACCGCGTC-3′
Canine BRCA2 nested PCR	5′-AGAGCGGCACCTCGGAAGG-3′	5′-TTTTATCTACAATATTACTCCAGTGCTTGG-3′
Human BRCA2 first PCR	5′-gtcaagcttGTGGCGCGAGCTTCTGAAAC-3′	5′-gggctcgagCTGCTTTGTTGCAGCGTGTC-3′
Human BRCA2 nested PCR	5′-GAACTGCACCTCTGGAGCG-3′	5′-CCTCCAATGCTTGGTAAATAAGT-3′
For quantitative RT-PCR		
Canine BRCA2 exon 26–7	5′-GACTATGCTTCAGAGCCACACACAG-3′	5′-GAAGTCATTTGGGTTGATCCAGGTA-3′
Canine BRCA2 exon 1–2	5′-GTCAGCTTTCTGGCCGAAGT-3′	5′-GTAAATAACTCGCCTTCCGAG-3′
Canine RPS18	5′-ATAGCCTTTGCCATCACAGCAATTA-3′	5′-TTGGTGAGATCGATGTGTCTGCTTTC-3′
Human BRCA2 exon 24–5	5′-AGGACTTGCCCCTTTCGTCTA-3′	5′-TGCAGCAATTAACATATGAGG-3′
Human BRCA2 exon 1–2	5′-CTCTGGAGCGGACTTATTTACC-3′	5′-AATGTTGGCCTCTCTTTGGA-3′
Human RPS18	5′-TGCGAGTACTCAACACCAAC-3′	5′-AGCATATCTTCGGCCCACAC-3′
Firefly luciferase	5′-GATGTACACGTTCGTCACATCTC-3′	5′-GACACCTTTAGGCAGACCAGTAG-3′
Human BRCA2 exon 27	5′-TCATGCCTGTAATCCCAACA-3′	5′-AAGATGGGGGTCTCCCTATG-3′

### Luciferase assay

Approximately 2 × 10^5^ HeLa cells were transiently co-transfected with 20 ng canine or human *BRCA2* 5′ UTR and firefly luciferase cloned pcDNA3 (Invitrogen) or pGL4 (Promega) vectors and 10 ng pRL-TK (Promega) using FuGENE HD Transfection Reagent (Roche Diagnostics, Basel, Switzerland). Forty-eight hours after transfection, the cells were lysed in Passive Lysis Buffer (Promega). Firefly and *Renilla* luciferase activities were measured using the Dual-Luciferase Reporter Assay Kit (Promega) according to the manufacturer’s instructions. Transfection efficiency was normalized by measuring *Renilla* luciferase activity.

### Quantitative real-time PCR

RNA was extracted from the transfected cells using the CellAmp™ Direct RNA Prep Kit for RT-PCR (Real Time) (Takara, Japan), and cDNA synthesis was performed as described above. Genomic DNA was also prepared using a Gentra Puregene Cell Kit (Qiagen, Hilden, Germany). Quantitative real-time polymerase chain reaction (qPCR) was carried out in a StepOnePlus Real-Time PCR System (Life Technologies, Carlsbad, CA, USA) using a KAPA SYBR FAST qPCR Kit (Kapa Biosystems, Wilmington, MA, USA) and 200 nM of each primer (Table 1). The PCR cycling conditions were as follows: an initial denaturation of 95 °C for 20 s followed by 40 PCR cycles of 95 °C for 3 s and 60 °C for 30 s. Melting curves were generated at the end of each real-time PCR run to ensure that a single specific product was amplified. Each sample was evaluated in triplicate. The housekeeping gene canine or human *RPS18* was used as an internal reference for normalization. The human *BRCA2* exon 27 region and firefly luciferase from genomic DNA samples were used to normalize the transfection efficiency.

### Treatment with ionizing radiation

Approximately 2 × 10^5^ HeLa cells were seeded into 4-well plates. After 24 h, irradiation of HeLa cells in Dulbecco’s modified Eagle’s medium supplemented with 10% fetal bovine serum was carried out in a MX-80Labo (mediXtec Japan, Japan) for a total dose of 10 Gy (dose rate; 0.67 Gy/min) at room temperature. Irradiated cells were incubated at 37 °C under humidified 5% CO_2_–95% air and after the indicated time, the cells were harvested and the RNA was extracted.

### Statistical analysis

To compare two groups, statistical analyses were performed using an F test followed by Student’s *t*-test. To compare more than three groups, one-way analysis of variance with Dunnett’s test or F test followed by Student’s *t*-test correcting with Holm’s method was performed.

## Supplementary Information

**Additional file 1: Fig. S1.** Messenger RNA base sequences encoded by exon 1-intron 1-exon 2 region of canine and human *BRCA2*. Bold characters indicate possible start codons and bold AUG with box indicates actual start sites in exon 2. Underlined characters indicate the GU-AG sites for splicing.

## Data Availability

The datasets used and/or analyzed during the current study are available from the corresponding author on reasonable request.

## Abbreviations

BRCA2: Brest cancer 2, early onset
HR: Homologous recombination
RAD51: RAD51 recombinase
DMC1: DNA meiotic recombinase 1
ZAR1L: Zygote arrest 1 like
PARP1: Poly (ADP-ribose) polymerase 1
USP11: Ubiquitin specific peptidase 11
RT-PCR: Reverse-transcription-polymerase chain reaction
PCR: Polymerase chain reaction
CMV: Cytomegalovirus
H3K27Ac: Histone H3 acetylated lysine 27
UCSC: University of California Santa Cruz

## References

[1] Fradet-Turcotte A, Sitz J, Grapton D, Orthwein A (2016). BRCA2 functions: from DNA repair to replication fork stabilization. Endocr Relat Cancer.

[2] Rivera P, Melin M, Biagi T, Fall T, Haggstrom J, Lindblad-Toh K (2009). Mammary tumor development in dogs is associated with BRCA1 and BRCA2. Cancer Res.

[3] Scully R, Livingston DM (2000). In search of the tumour-suppressor functions of BRCA1 and BRCA2. Nature Nature Publishing Group.

[4] Yoshikawa Y, Morimatsu M, Ochiai K, Ishiguro-Oonuma T, Wada S, Orino K (2015). Reduced canine BRCA2 expression levels in mammary gland tumors. BMC Vet Res BioMed Central.

[5] Yoshikawa Y, Morimatsu M, Ochiai K, Nagano M, Yamane Y, Tomizawa N (2005). Analysis of genetic variations in the exon 27 region of the canine BRCA2 locus. J Vet Med Sci.

[6] Yoshikawa Y, Morimatsu M, Ochiai K, Nagano M, Tomioka Y, Sasaki N (2008). Novel variations and loss of heterozygosity of BRCA2 identified in a dog with mammary tumors. Am J Vet Res.

[7] Tan SLW, Chadha S, Liu Y, Gabasova E, Perera D, Ahmed K (2017). A class of environmental and endogenous toxins induces BRCA2 Haploinsufficiency and genome instability. Cell Elsevier Inc.

[8] Magwood AC, Malysewich MJ, Cealic I, Mundia MM, Knapp J, Baker MD (2013). Endogenous levels of Rad51 and Brca2 are required for homologous recombination and regulated by homeostatic re-balancing. DNA Repair.

[9] Arnold K, Kim M-K, Frerk K, Edler L, Savelyeva L, Schmezer P (2006). Lower level of BRCA2 protein in heterozygous mutation carriers is correlated with an increase in DNA double strand breaks and an impaired DSB repair. Cancer Lett.

[10] Martinez JS, von Nicolai C, Kim T, Ehlén Å, Mazin AV, Kowalczykowski SC (2016). BRCA2 regulates DMC1-mediated recombination through the BRC repeats. Proc Natl Acad Sci USA.

[11] Sharan SK, Pyle A, Coppola V, Babus J, Swaminathan S, Benedict J (2004). BRCA2 deficiency in mice leads to meiotic impairment and infertility. Development..

[12] Daum H, Peretz T, Laufer N (2018). BRCA mutations and reproduction. Fertil Steril.

[13] Lin W, Titus S, Moy F, Ginsburg ES, Oktay K (2017). Ovarian aging in women with BRCA Germline mutations. J Clin Endocrinol Metab.

[14] Zhoucun A, Zhang S, Yang Y, Ma Y, Zhang W, Lin L (2006). The common variant N372H in BRCA2 gene may be associated with idiopathic male infertility with azoospermia or severe oligozoospermia. Eur J Obstet Gynecol Reprod Biol.

[15] Wu K, Jiang S-W, Couch FJ (2003). p53 mediates repression of the BRCA2 promoter and down-regulation of BRCA2 mRNA and protein levels in response to DNA damage. J Biol Chem.

[16] Misra S, Sharma S, Agarwal A, Khedkar SV, Tripathi MK, Mittal MK (2010). Cell cycle-dependent regulation of the bi-directional overlapping promoter of human BRCA2/ZAR2 genes in breast cancer cells. Mol Cancer.

[17] Wang J, Bian C, Li J, Couch FJ, Wu K, Zhao RC (2008). Poly (ADP-ribose) polymerase-1 down-regulates BRCA2 expression through the BRCA2 promoter. J Biol Chem.

[18] Wu K, Jiang SW, Thangaraju M, Wu G, Couch FJ (2000). Induction of the BRCA2 promoter by nuclear factor-kappa B. J Biol Chem.

[19] Choi YE, Pan Y, Park E, Konstantinopoulos P, De S, D'Andrea A (2014). MicroRNAs down-regulate homologous recombination in the G1 phase of cycling cells to maintain genomic stability. Elife..

[20] Song L, Dai T, Xie Y, Wang C, Lin C, Wu Z (2012). Up-regulation of miR-1245 by c-myc targets BRCA2 and impairs DNA repair. J Mol Cell Biol.

[21] Schoenfeld AR, Apgar S, Dolios G, Wang R, Aaronson SA (2004). BRCA2 is ubiquitinated in vivo and interacts with USP11, a deubiquitinating enzyme that exhibits prosurvival function in the cellular response to DNA damage. Mol Cell Biol.

[22] Hinnebusch AG, Ivanov IP, Sonenberg N (2016). Translational control by 5′-untranslated regions of eukaryotic mRNAs. Science. Am Assoc Advancement Sci.

[23] Shkreta L, Chabot B (2015). The RNA splicing response to DNA damage. Biomolecules..

[24] Sprung CN, Li J, Hovan D, McKay MJ, Forrester HB (2011). Alternative Transcript Initiation and Splicing as a Response to DNA Damage. Bernhard EJ, editor. PLoS ONE. Public Libr Sci.

[25] Magwood AC, Mundia MM, Baker MD. High levels of wild-type BRCA2 suppress homologous recombination. J Mol Biol. 2012;421:38–53. 10.1016/j.jmb.2012.05.007 22579622

[26] Tripathi MK, Misra S, Khedkar SV, Hamilton N, Irvin-Wilson C, Sharan C (2005). Regulation of BRCA2 gene expression by the SLUG repressor protein in human breast cells. J Biol Chem.

[27] Yoshikawa Y, Morimatsu M, Ochiai K, Okuda K, Taoda T, Chikazawa S (2012). Establishment of a PCR analysis method for canine BRCA2. BMC Res Notes.

